# An inflammation-based model for identifying severe acute pancreatitis: a single-center retrospective study

**DOI:** 10.1186/s12876-024-03148-4

**Published:** 2024-02-05

**Authors:** Xiaotong Li, Yiyan Zhang, Weiwei Wang, Yao Meng, Huimin Chen, Guiyang Chu, Hongyu Li, Xingshun Qi

**Affiliations:** 1 Department of Gastroenterology, General Hospital of Northern Theater Command, No. 83 Wenhua Road, Shenyang, 110840 Liaoning Province China; 2 Postgraduate College, China Medical University, Shenyang, China; 3 Postgraduate College, Liaoning University of Traditional Chinese Medicine, Shenyang, China; 4 Postgraduate College, Dalian Medical University, Dalian, China; 5 Information Section of Medical Security Center, General Hospital of Northern Theater Command, Shenyang, China

**Keywords:** Acute pancreatitis, Severe acute pancreatitis, Inflammatory index, Identification, Model

## Abstract

**Background and aims:**

Severe acute pancreatitis (SAP) is potentially lethal. Considering the role of inflammation in the progression of acute pancreatitis (AP), this study aims to develop a model based on inflammatory indexes for identifying the presence of SAP.

**Methods:**

Overall, 253 patients with AP who were consecutively admitted between July 2018 and November 2020 were screened, of whom 60 had SAP. Systemic immune-inflammation index (SII), neutrophil-to-lymphocyte ratio (NLR), platelet-to-lymphocyte ratio (PLR), lymphocyte-to-monocyte ratio (LMR), neutrophil-to-platelet ratio (NPR), systemic inflammation response index (SIRI), platelet-to-albumin ratio (PAR), C-reactive protein-to-albumin ratio (CAR), C-reactive protein-to-lymphocyte ratio (CLR), and triglyceride glucose (TyG) index were calculated. Multivariate logistic regression analyses were performed to identify independent risk factors of SAP. Then, inflammation-based models were established. Receiver operating characteristics (ROC) curve analyses were performed. Area under ROC curve (AUROC) was calculated.

**Results:**

Diabetes mellitus, fatty liver, high white blood cell count (WBC), C-reactive protein (CRP), red blood cell distribution width (RDW), procalcitonin (PCT), SII, NLR, NPR, CAR, CLR, and TyG index, and a low LMR were significantly associated with SAP. Considering the collinearity among these variables, 10 multivariate logistic regression analyses were separately performed. Finally, four independent inflammation-based models were established. Of them, the best one, which was calculated as follows: 1.204*fatty liver (yes = 1; no = 0) + 0.419*PCT + 0.005*CLR - 2.629, had an AUROC of 0.795 with a specificity of 73.4% and a sensitivity of 71.7%.

**Conclusion:**

The inflammation-based model consisting of fatty liver, PCT, and CLR has a good diagnostic performance for SAP.

**Supplementary Information:**

The online version contains supplementary material available at 10.1186/s12876-024-03148-4.

## Introduction

Acute pancreatitis (AP) is an inflammatory disorder triggered by pancreatic enzyme infiltration [1] with an incidence of 34 cases per 100,000 person-years around the world [2, 3]. Its main clinical presentation includes severe abdominal pain with or without nausea, vomiting, and fever [4]. It is mostly mild and may resolve within a few days [5]. However, about 20% of the patients progress to severe acute pancreatitis (SAP), which is characterized by systemic inflammatory response syndrome (SIRS) with single or multiple organ failure [5, 6], with a mortality of up to 50% [7, 8]. Identification of the patients who are at a high risk of developing SAP is necessary for predicting their outcomes and guiding the treatment strategy [9].

Inflammation is critical for the development and progression of SAP [10]. During the course of SAP, excessive inflammatory mediators are released, inducing inflammatory cascade reaction, ultimately causing bacterial translocation and secondary injuries of distant tissues and organs [11, 12]. Thus, it seems to be reasonable that inflammatory indexes, such as neutrophil to lymphocyte ratio (NLR) and lymphocyte to monocyte ratio (LMR), can predict the probability of SAP [13, 14]. But their performance was very limited, probably because only a single index was employed in previous studies. Tanoğlu et al. also suggested that NLR alone may not truly reflect the severity of AP due to the possible influencing factors, such as other diseases [15]. Subsequently, some researchers have attempted to combine various inflammatory indexes for the prediction of SAP. Kaplan et al. found a similar predictive performance of the platelet to lymphocyte ratio (PLR)-NLR combination with other scoring systems for determining the prognosis of AP patients. But there is collinearity between PLR and NLR [16]. Zhu et al. also reported a good predictive value of a combination of NLR, procalcitonin (PCT), and modified computerized tomography severity index (MCTSI) for infected pancreatic necrosis, a form of SAP [17]. But it requires the results of imaging examinations except for inflammatory indexes. Herein, we aimed to develop a model for identifying SAP by combining various inflammatory indexes.

## Methods

### Study design

The retrospective study was performed according to the Declaration of Helsinki and was approved by the Medical Ethical Committee of the General Hospital of Northern Theater Command (approval number: Y2023–120). Written informed consent was waived by the Medical Ethical Committee of the General Hospital of Northern Theater Command due to its retrospective nature. We reviewed the medical records of all patients who were diagnosed with AP and consecutively admitted to the General Hospital of Northern Theater Command between July 4, 2018 and November 20, 2020 from the Information Section of Medical Security Center. The exclusion criteria were as follows: (1) age < 18 or > 80 years; (2) medical records cannot be reviewed in detail; (3) the interval between onset of symptoms and admission was more than seven days; (4) the hospital stay was less than 5 days; (5) co-existing severe trauma or pregnancy; (6) co-existing chronic pancreatitis; and (7) co-existing viral infection or rheumatic diseases.

### Data collection

Patients’ demographics (i.e., age and gender), comorbidities (i.e., diabetes mellitus, hypertension, hypertriglyceridemia, and fatty liver), history of smoking and alcohol drinking, history of AP, blood tests at admission (i.e., white blood cell count [WBC], lymphocyte count, platelet count, neutrophil count, monocyte count, C-reactive protein [CRP], red blood cell distribution width [RDW], platelet distribution width [PDW], and PCT), interval between onset of symptom and admission, length of hospital stay, and hospitalization expenses were retrieved from the inpatients’ electronic medical records. Several inflammatory indexes, including systemic immune inflammation index (SII), NLR, PLR, LMR, neutrophil to platelet ratio (NPR), systemic inflammation response index (SIRI), platelet to albumin ratio (PAR), CRP to albumin ratio (CAR), CRP to lymphocyte ratio (CLR), and triglyceride glucose (TyG) index were calculated. SII was calculated as the neutrophil counts (10^9^/L) multiplied by the platelet counts (10^9^/L) and divided by the lymphocyte counts (10^9^/L) [18]. NLR was calculated as the neutrophil counts (10^9^/L) divided by the lymphocyte counts (10^9^/L) [19]. PLR was calculated as the platelet counts (10^9^/L) divided by the lymphocyte counts (10^9^/L) [20]. LMR was calculated as the lymphocyte counts (10^9^/L) divided by the monocyte counts (10^9^/L) [21]. NPR was calculated as the neutrophil counts (10^9^/L) multiplied by 1000 and divided by the platelet counts (10^9^/L) [22]. SIRI was calculated as the neutrophil counts (10^9^/L) multiplied by the monocyte counts (10^9^/L) and divided by the lymphocyte counts (10^9^/L) [23]. PAR was calculated as the platelet counts (10^9^/L) divided by the albumin levels (g/L) [24]. CAR was calculated as the CRP levels (mg/L) divided by the albumin levels (g/L) [25]. CLR was calculated as the CRP levels (mg/L) divided by the lymphocyte counts (10^9^/L) [26]. TyG index was calculated as Ln (the triglycerides [mg/dL] multiplied by the blood glucose [mg/dL]/2) [27].

### Group and definition

In the present study, the patients were classified into SAP and N-SAP group according to the revised Atlanta criteria and Ranson and BISAP scoring systems. According to the revised Atlanta criteria, AP is classified as follows [28]: (1) mild acute pancreatitis (MAP), which is defined if patients have neither local complications nor organ failure; (2) moderately acute pancreatitis (MSAP), which is defined if patients have transient organ failure (< 48 h) and/or local complications; (3) SAP, which is defined if patients have persistent organ failure (≥ 48 h) with or without local complications. Ranson score is calculated based on 11 variables: age > 55 years, WBC > 16,000/μL, lactate dehydrogenase> 350 U/L, aspartate transaminase> 250 U/L, and blood glucose> 200 mg/dL at admission, and fall in hematocrit> 10%, increase in blood urea nitrogen (BUN) > 5 mg/dL, calcium< 8 mg/dL, PaO_2_ < 60 mmHg, base deficit> 4 mEq/L, and fluid loss> 6 L within 48 h after admission [29]. BISAP score is calculated based on five variables: BUN> 25 mg/dL, impaired mental status, SIRS, age > 60 years, and radiographic evidence of pleural effusion within the first 24 hours after admission [30]. Ranson or BISAP score ≥ 3 is defined as SAP; otherwise, N-SAP is considered. Because not all of our patients had the data at 24 h or 48 h, we selected the value reflecting the most severe clinical condition during their hospitalizations.

### Statistical analyses

Continuous variables were presented as mean ± standard deviation (SD) and median with range. If the variables followed normal distribution, their differences between groups would be evaluated by independent sample T-test; otherwise, their differences between groups would be evaluated by Mann-Whitney U test. Categorical variables were presented as frequency with percentage. Differences between groups were evaluated by Chi-squared test or Fisher’s exact test. Statistically significant factors in the univariate logistic regression analyses were included in the multivariate logistic regression analyses. Multivariate logistic regression models were established after eliminating the factors with collinearity. The discrimination of the models was evaluated and compared by calculating the area under the receiver operating characteristic curve (AUROC). The concordance index (c-index) was calculated, and the calibration curve was plotted by bootstrapping with 1000 resamples to evaluate the accuracy and conformity of the models. SPSS 26.0, R 4.2.2, and GraphPad Prism 8.0.1 statistical software were used for the data analyses. A two-tailed *P* < 0.05 was statistically significant.

## Results

### Characteristics of patients

**Fig. 1:**
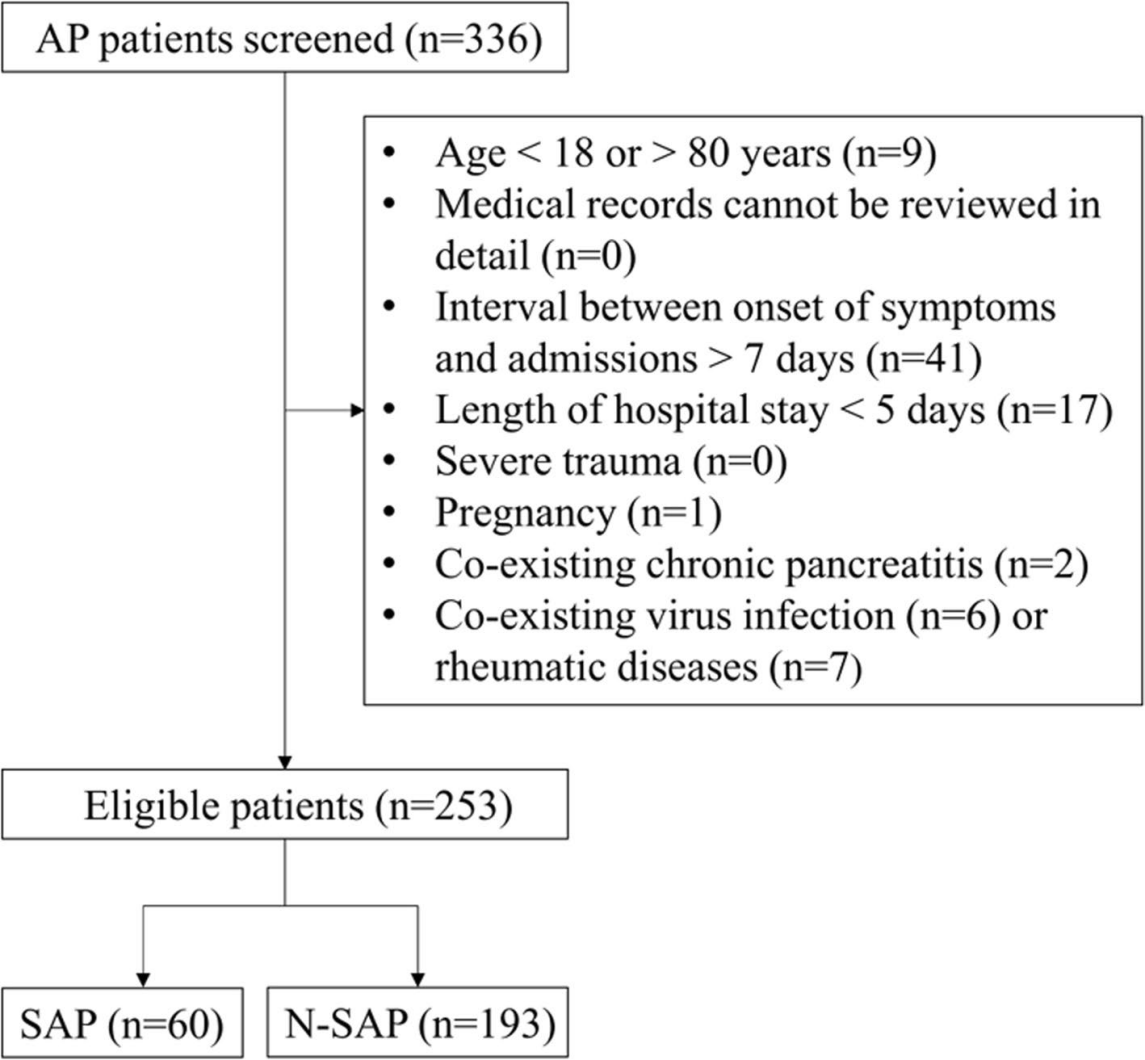
A flow chart of patients’ selection

Initially, 336 AP patients were screened. Finally, 253 patients were included, of whom 60 and 193 were assigned to SAP and N-SAP group, respectively (Fig. 1). Baseline characteristics of the patients were shown in Table 1. The mean age was 45.98 ± 0.89 years, and 64.0% of the patients were male. The mean length of hospital stay was 12.02 ± 0.35 days, the mean interval between onset of symptom and admission was 1.50 ± 0.11 days, and the mean hospitalization expense was 27,160.88 ± 1279.54 yuan.

**Table 1:** Baseline characteristics of the AP patients

Variables	No. Pts	Mean ± SD, Median (range) or Frequency (percentage)
Age (years)	253	45.98 ± 0.89 44.20 (18.50–78.50)
Male (%)	253	162 (64.0%)
Smoking (%)	253	101 (39.9%)
Alcohol (%)	253	71 (28.1%)
History of AP (%)	253	83 (32.8%)
***Comorbidities***		
Diabetes mellitus (%)	253	70 (27.7%)
Hypertriglyceridemia (%)	253	134 (53.0%)
Hypertension (%)	253	55 (21.7%)
Fatty liver (%)	253	143 (56.5%)
***Laboratory parameters***		
WBC (10^9^/L)	251	12.02 ± 0.29 11.60 (2.40–27.10)
CRP (mg/L)	205	86.48 ± 6.08 57.23 (0.50–329.12)
RDW (%)	251	13.21 ± 0.08 13.10 (4.19–22.60)
PDW (%)	251	16.89 ± 0.05 16.70 (15.30–19.50)
PCT (ng/mL)	186	0.69 ± 0.13 0.12 (0.02–18.62)
***Inflammatory indexes***		
SII	251	2522.35 ± 292.09 1737.00 (102.21–67,125.00)
NLR	251	10.35 ± 0.87 7.41 (0.71–179.00)
PLR	251	227.38 ± 16.92 175.76 (0.12–3750.00)
LMR	249	4.15 ± 1.06 2.40 (0.06–258.00)
NPR	251	43.84 ± 1.27 40.66 (7.86–111.64)
SIRI	251	7.91 ± 1.40 4.02 (0.00–304.30)
PAR	225	8.57 ± 1.69 5.86 (2.58–318.00)
CAR	189	2.49 ± 0.19 1.56 (0.01–11.58)
CLR	207	94.27 ± 10.21 41.17 (0.20–1296.10)
TyG index	228	2.58 ± 0.10 2.36 (−0.27–6.82)
***SAP (%)***	253	60 (23.72%)
***Length of hospital stay (days)***	253	12.03 ± 0.35 11.00 (5.00–42.00)
***Hospitalization expense (yuan)***	253	27,160.88 ± 1279.54 20969.06 (2381.87–141,640.28)
***Interval between symptom onset and admission***	253	1.50 ± 0.11 1.00 (0.00–7.00)

*Abbreviations: SAP* severe acute pancreatitis,  *AP* acute pancreatitis, *No. Pts* number of patients, *CRP* C-reactive protein, *RDW* red blood cell distribution width, *PDW* platelet distribution width, *PCT* procalcitonin, *SII* systemic immune-inflammation index, *NLR* neutrophil to lymphocyte ratio, *PLR* platelet to lymphocyte ratio, *LMR* lymphocyte to monocyte ratio, *NPR* neutrophil to platelet ratio, *SIRI* systemic inflammatory response index, *PAR* platelet to albumin ratio, *CAR* C-reactive protein to albumin ratio, *CLR* C-reactive protein to lymphocyte ratio, *TyG index* triglyceride-glucose index

### Difference between SAP and N-SAP groups

Gender, diabetes mellitus, and fatty liver were significantly different between the two groups. WBC, CRP, RDW, PDW, PCT, SII, NLR, PLR, NPR, SIRI, PAR, CAR, CLR, TyG index, length of hospital stay, and hospitalization expense were significantly higher in the SAP group than the N-SAP group. LMR was significantly lower in the SAP group than the N-SAP group. Age, history of smoking, drinking, and AP, hypertriglyceridemia, hypertension, and interval between onset of symptom and admission were statistically similar between them (Table 2).

**Table 2:** Comparison between SAP and N-SAP groups

Variables	No. Pts	SAP	No. Pts	N-SAP	***P*** value
Age (years)	60	48.00 ± 2.10 44.55 (18.50–77.20)	193	45.36 ± 0.96 43.80 (19.70–78.50)	0.341
Male (%)	60	32 (53.33%)	193	130 (67.36%)	**0.048**
Smoking (%)	60	18 (30.00%)	193	83 (43.01%)	0.072
Alcohol (%)	60	17 (28.33%)	193	54 (27.98%)	0.957
History of AP (%)	60	15 (25.00%)	193	68 (35.23%)	0.140
***Comorbidities***					
Diabetes mellitus (%)	60	25 (41.67%)	193	45 (23.32%)	**0.006**
Hypertriglyceridemia (%)	60	37 (61.67%)	193	97 (50.26%)	0.122
Hypertension (%)	60	15 (25.00%)	193	40 (20.73%)	0.483
Fatty liver (%)	60	47 (78.33%)	193	96 (49.74%)	**< 0.001**
***Laboratory parameters***					
WBC (10^9^/L)	60	14.00 ± 0.60 13.55 (4.80–27.10)	191	11.40 ± 0.32 11.30 (2.40–25.40)	**< 0.001**
CRP (mg/L)	52	141.80 ± 13.12 148.53 (0.89–326.60)	153	67.68 ± 6.13 35.47 (0.50–329.12)	**< 0.001**
RDW (%)	60	13.63 ± 0.19 13.30 (11.90–22.60)	191	13.08 ± 0.08 13.00 (4.19–18.00)	**0.001**
PDW (%)	60	17.13 ± 0.11 16.95 (15.90–19.50)	191	16.82 ± 0.06 16.60 (15.30–19.50)	**0.008**
PCT (ng/mL)	53	1.50 ± 0.39 0.37 (0.03–18.62)	133	0.36 ± 0.08 0.10 (0.02–8.10)	**< 0.001**
***Inflammatory indexes***					
SII	60	4222.55 ± 1124.23 2320.95 (231.69–67,125.00)	191	1988.25 ± 134.00 1440.75 (102.21–12,082.76)	**< 0.001**
NLR	60	15.67 ± 3.05 9.29 (1.46–179.00)	191	8.68 ± 0.58 7.05 (0.71–54.45)	**< 0.001**
PLR	60	304.41 ± 61.08 220.54 (45.43–3750.00)	191	203.18 ± 10.88 168.42 (0.12–1395.24)	**0.007**
LMR	59	2.10 ± 0.16 1.70 (0.06–5.67)	190	4.79 ± 1.38 2.50 (0.09–258.00)	**< 0.001**
NPR	60	51.66 ± 2.60 48.85 (22.89–111.64)	191	41.38 ± 1.42 38.91 (7.86–101.35)	**0.001**
SIRI	60	14.03 ± 5.10 6.08 (0.00–304.30)	191	5.98 ± 0.88 3.47 (0.00–114.89)	**< 0.001**
PAR	57	6.81 ± 0.33 6.71 (2.90–18.97)	168	9.17 ± 2.26 5.66 (2.58–318.00)	**0.012**
CAR	50	4.22 ± 0.41 3.87 (0.02–11.58)	139	1.87 ± 0.19 0.98 (0.01–9.46)	**< 0.001**
CLR	53	191.60 ± 31.57 130.40 (0.25–1296.10)	154	60.77 ± 6.59 25.35 (0.20–442.00)	**< 0.001**
TyG index	58	2.96 ± 0.22 3.12 (−0.27–5.33)	170	2.45 ± 0.12 2.20 (−0.20–6.82)	**0.026**
***Length of hospital stay (days)***	60	15.03 ± 0.82 14.00 (6.00–36.00)	193	11.09 ± 0.36 10.00 (5.00–42.00)	**< 0.001**
***Hospitalization expense (yuan)***	60	47,228.68 ± 3521.65 41,001.90 (8368.46–141,640.28)	193	20,932.58 ± 766.37 18,684.38 (2381.87–63,805.67)	**< 0.001**
***Interval between symptom onset and admission***	60	1.33 ± 0.18 1.00 (0.00–6.00)	193	1.55 ± 0.13 1.00 (0.00–7.00)	0.778

*Abbreviations: SAP* severe acute pancreatitis, *AP* acute pancreatitis, *No. Pts* number of patients, *CRP* C-reactive protein, *RDW* red blood cell distribution width, *PDW* platelet distribution width, *PCT* procalcitonin, *SII* systemic immune-inflammation index, *NLR* neutrophil to lymphocyte ratio, *PLR* platelet to lymphocyte ratio, *LMR* lymphocyte to monocyte ratio, *NPR* neutrophil to platelet ratio, *SIRI* systemic inflammatory response index, *PAR* platelet to albumin ratio, *CAR* C-reactive protein to albumin ratio, *CLR* C-reactive protein to lymphocyte ratio, *TyG index* triglyceride-glucose index

### Inflammatory index models

**Fig. 2:**
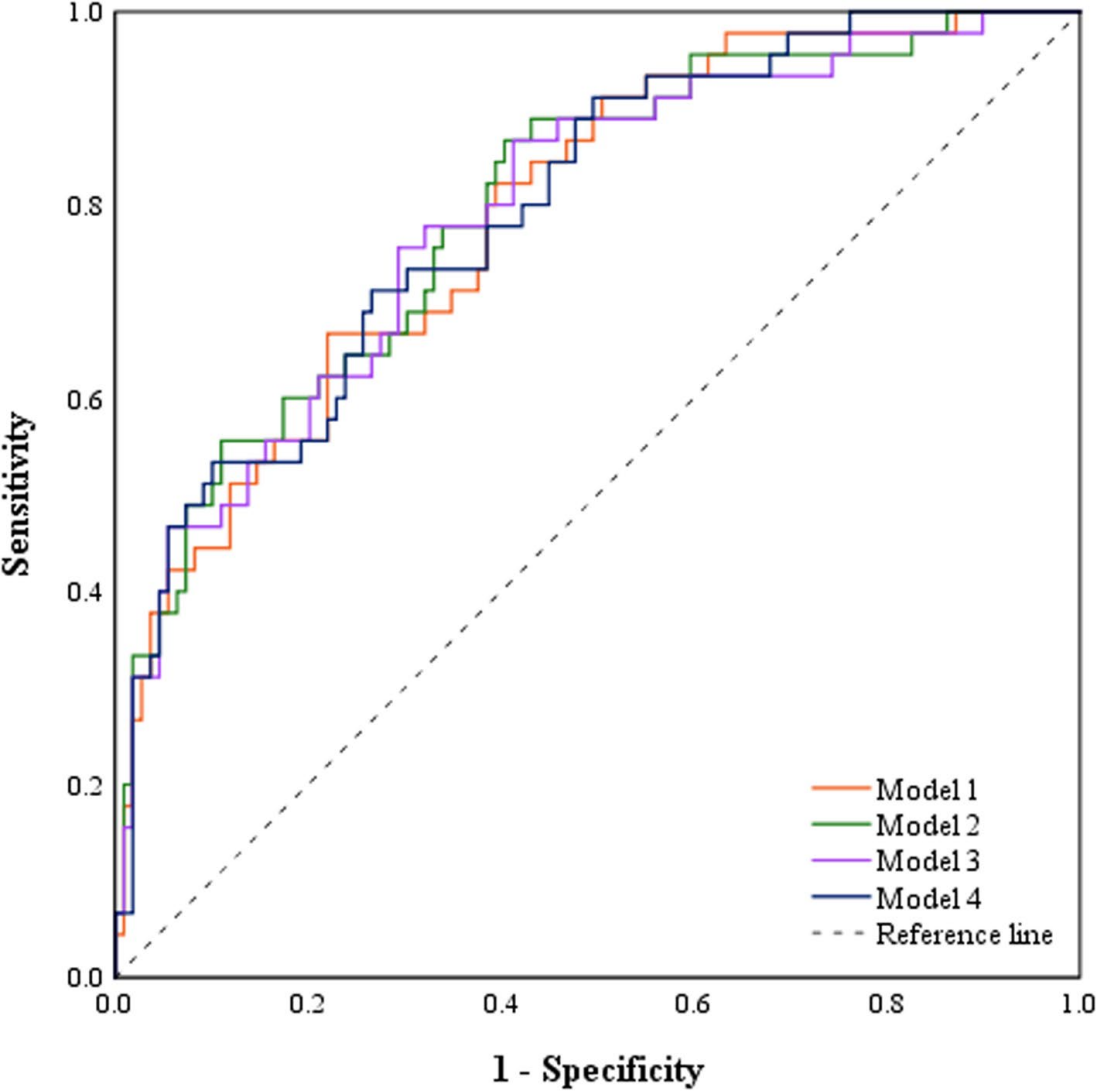
Receiver operating characteristic (ROC) curves of four inflammation-based models. Model 1 comprises fatty liver, PCT, and LMR. Model 2 comprises fatty liver, PCT, and SII. Model 3 comprises fatty liver, PCT, and NLR. Model 4 comprises fatty liver, PCT, and CLR

**Fig. 3:**
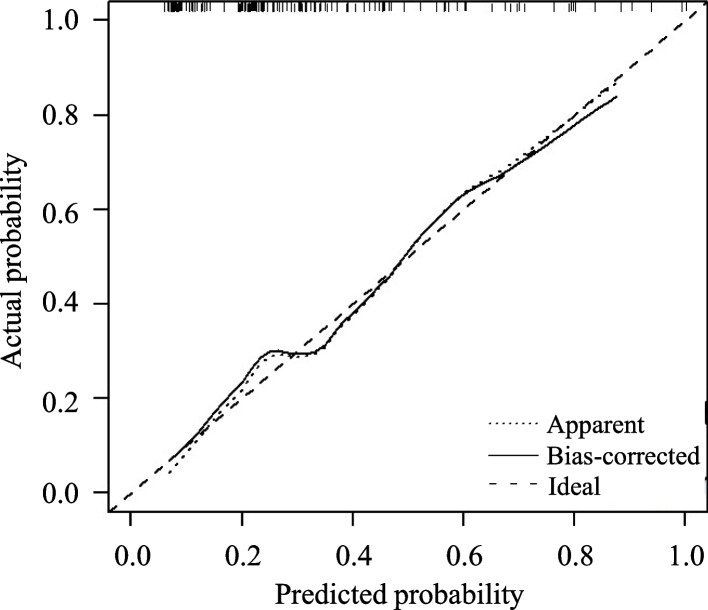
Calibration curve of the model 4: fatty liver (yes = 1 and no = 0) × 1.204+ PCT × 0.419 + CLR × 0.005–2.629

Univariate logistic regression analyses demonstrated that fatty liver, high WBC, CRP, RDW, PCT, SII, NLR, NPR, CAR, CLR, and TyG index, and a low LMR were significantly associated with the presence of SAP (Table 3). Considering the collinearity among these inflammatory indexes, 10 multivariate logistic regression analyses were performed (Supplementary Figs. 1–10). Finally, four models for the identification of SAP were established (Table 4). Their AUROCs were 0.771–0.795 (Fig. 2). Of them, the model 4 had the best discrimination ability (Fig. 2). The c-index was 0.795, and the calibration curve was close to the ideal diagonal line, indicating a good fit (Fig. 3).

**Table 3:** Univariate logistic regression analysis for predictors of SAP

Variables	OR (95% CI)	*P* value
Age (years)	1.013 (0.993–1.034)	0.205
Gender (male versus female)	1.806 (1.001–3.256)	0.050
Smoking (yes versus no)	0.568 (0.305–1.057)	0.074
Alcohol (yes versus no)	1.018 (0.535–1.937)	0.957
History of AP (yes versus no)	0.613 (0.318–1.179)	0.143
Diabetes mellitus (yes versus no)	2.349 (1.274–4.333)	0.006
Hypertriglyceridemia (yes versus no)	1.592 (0.881–2.878)	0.124
Hypertension (yes versus no)	1.275 (0.646–2.517)	0.484
Fatty liver (yes versus no)	3.653 (1.858–7.181)	**< 0.001**
WBC (10^9^/L)	1.129 (1.058–1.205)	**< 0.001**
CRP (mg/L)	1.010 (1.006–1.013)	**< 0.001**
RDW (%)	1.478 (1.117–1.955)	**0.006**
PDW (%)	1.511 (1.086–2.103)	**0.014**
PCT (ng/mL)	1.849 (1.322–2.587)	**< 0.001**
SII	1.000 (1.000–1.000)	**0.001**
NLR	1.050 (1.018–1.084)	**0.002**
PLR	1.002 (1.000–1.003)	0.055
LMR	0.673 (0.536–0.845)	**0.001**
NPR	1.025 (1.010–1.040)	**0.001**
SIRI	1.019 (0.998–1.041)	0.077
PAR	0.994 (0.973–1.015)	0.582
CAR	1.402 (1.226–1.604)	**< 0.001**
CLR	1.008 (1.005–1.011)	**< 0.001**
TyG index	1.227 (1.012–1.488)	**0.037**
Interval between symptom onset and admission	0.925 (0.774–1.105)	0.389

*Abbreviations: SAP* severe acute pancreatitis, *OR* odds ratio, *CI* confidence interval, *CRP* C-reactive protein, *RDW* red blood cell distribution width, *PDW* platelet distribution width, *PCT* procalcitonin, *SII* systemic immune-inflammation index, *NLR* neutrophil to lymphocyte ratio, *PLR* platelet to lymphocyte ratio, *LMR* lymphocyte to monocyte ratio, *NPR* neutrophil to platelet ratio, *SIRI* systemic inflammatory response index, *PAR* platelet to albumin ratio, *CAR* C-reactive protein to albumin ratio, *CLR* C-reactive protein to lymphocyte ratio, *TyG index* triglyceride-glucose index

**Table 4:** Multivariate logistic regression models for identifying SAP

Model	Equation	AUROC (95% CI)	Cutoff value	Sensitivity	Specificity
***Model 1***	Fatty liver (yes = 1 and no = 0) × 1.428 + PCT × 0.651 - LMR × 0.303–1.623	0.777 (0.704–0.849)	0.329	0.635	0.782
***Model 2***	Fatty liver (yes = 1 and no = 0) × 1.332 + PCT × 0.586+ SII × 0.0002–2.833	0.780 (0.708–0.852)	0.228	0.906	0.526
***Model 3***	Fatty liver (yes = 1 and no = 0) × 1.344+ PCT × 0.583 + NLR × 0.045–2.801	0.771 (0.697–0.845)	0.264	0.755	0.654
***Model 4***	Fatty liver (yes = 1 and no = 0) × 1.204+ PCT × 0.419 + CLR × 0.005–2.629	0.795 (0.720–0.869)	0.287	0.717	0.734

*Abbreviations: SAP* severe acute pancreatitis, *PCT* procalcitonin, *LMR* lymphocyte to monocyte ratio, *SII* systemic immune-inflammation index, *NLR* neutrophil to lymphocyte ratio, *CLR* C-reactive protein to lymphocyte ratio, *CI* confidence interval, *AUROC* area under the receiver operating characteristic curve

## Discussion

In the present study, four inflammation-based models for identifying the presence of SAP have been established, of which one, consisting of fatty liver, PCT, and CLR, has a superior diagnostic performance with an accuracy of 79.5%. Notably, the three components are easily obtained through routine blood tests, allowing to assess the severity of AP rapidly.

CLR derives from CRP and lymphocyte. It was widely used to predict the prognosis in many diseases, such as oral cavity squamous cell carcinoma, pancreatic cancer, and colorectal cancer [26, 31, 32]. In our patients, CLR was positively associated with the risk of SAP. The possible reasons are as follows. First, CRP, an acute-phase protein, elevates dramatically during inflammation [33]. This is because that transcriptional induction of the CRP gene mainly occurs in response to an increase of inflammatory cytokines, especially IL-6 [34]. In SAP patients, IL-6 was excessively released [35, 36]. Besides, CRP is deposited at inflammatory sites and amplifies a pro-inflammatory response by a positive feedback loop [37]. Taken together, CRP rises in SAP patients. Second, lymphocyte counts are significantly reduced in SAP [38]. This may be due to the effects of endotoxins released from bacteria and cytokines on T lymphocyte reduction [39] and cytokines released from monocytes or endothelial cells on the apoptosis of peripheral lymphocytes [40].

PCT, a protein of 116 amino acids, is coded for by the calcitonin I (CALC-I) gene [41]. Serum PCT level is elevated in infectious diseases or conditions [42]. PCT was first found to be associated with the severity of infection in 1993 [43]. Later, it was demonstrated that PCT was a good predictor of short-term survival in patients with sepsis and pneumonia [44, 45]. Besides, high PCT level could predict the probability of SAP [46–48]. Similarly, our study found that PCT was an independent risk factor of SAP. This finding may be attributed to the fact that an increase of cytokines in SAP patients causes endotoxemia, inducing CALC-I expression in pancreas [49].

Previous studies also demonstrated the importance of other inflammatory indexes and prognostic scores for predicting the AP severity. Jain et al. reported that inflammatory indexes, including NLR, LMR, RDW, and PNI, were comparable to gold standard scoring systems for predicting the severity and mortality of AP [50]. Besides, CAR also has good predictive value in AP severity. It derives from CRP and albumin, which can be calculated rapidly and easily. Kiyak et al. compared CAR with traditional scores, and showed that CAR values were positively correlated with Balthazar score, and its AUC was higher than that of NLR and PLR in mortality prediction of AP [51]. However, their predictive values have not been confirmed in our study.

There were some limitations in our study. First, due to the retrospective nature of this study, some data could not be sufficiently extracted. Thus, we had to define SAP by meeting any of the three following criteria: the revised Atlanta criteria, BISAP score, or Ranson score. Besides, the extreme data obtained during hospitalizations, but not the data at baseline, was used to establish the model. Second, it should be noted that the interval between the onset of symptoms and laboratory assessment was different among our patients, but it could not be controlled due to the retrospective nature of our study. Third, this study was only performed at a single center. Therefore, the findings should be externally validated at other affiliations. Forth, only a relatively small number of AP patients were included. Thus, the findings should be validated in large-scale studies.

## Conclusion

Our study suggested an association of SAP with higher levels of SII, NLR, NPR, CAR, CLR, RDW, PDW, PCT, and TyG index, and a lower level of LMR. We developed an inflammation-based model comprising fatty liver, PCT, and CLR for identifying the presence of SAP with a good diagnostic ability. In future, multi-center studies should be conducted to validate these findings.

### Supplementary Information

**Additional file 1: Figure S1.** Forest plot showing the results of multivariate logistic regression analysis 1. **Figure S2.** Forest plot showing the results of multivariate logistic regression analysis 2. **Figure S3.** Forest plot showing the results of multivariate logistic regression analysis 3. **Figure S4.** Forest plot showing the results of multivariate logistic regression analysis 4. **Figure S5.** Forest plot showing the results of multivariate logistic regression analysis 5. **Figure S6.** Forest plot showing the results of multivariate logistic regression analysis 6. **Figure S7.** Forest plot showing the results of multivariate logistic regression analysis 7. **Figure S8.** Forest plot showing the results of multivariate logistic regression analysis 8. **Figure S9.** Forest plot showing the results of multivariate logistic regression analysis 9. **Figure S10.** Forest plot showing the results of multivariate logistic regression analysis 10.

## Data Availability

The datasets used and/or analyzed during the current study are available from.

## Abbreviations

AP: Acute pancreatitis
MAP: Mild acute pancreatitis
MSAP: Moderately severe acute pancreatitis
SAP: Severe acute pancreatitis
SIRS: Systemic inflammatory response syndrome
RDW: Red blood cell distribution width
PDW: Platelet distribution width
PCT: Procalcitonin
MCTSI: Modified computerized tomography severity index
BUN: Blood urea nitrogen
SII: Systemic immune inflammation index
NLR: Neutrophil to lymphocyte ratio
PLR: Platelet to lymphocyte ratio
LMR: Lymphocyte to monocyte ratio
NPR: Neutrophil to platelet ratio
SIRI: Systemic inflammation response index
PAR: Platelet to albumin ratio
CAR: C-reactive protein to albumin ratio
CLR: C-reactive protein to lymphocyte ratio
TyG index: Triglyceride glucose index
ROC curve: Receiver operating characteristics curve
AUROC: Area under receiver operating characteristics curve
c-index: Concordance index
SD: Standard deviation
MCV: Mean corpuscular volume
RBC: Red blood cells
CALC-I gene: calcitonin I gene
