# Supplementary material for: An inflammation-based model for identifying severe acute pancreatitis: a single-center retrospective study
Source: BMC Gastroenterol. 2024 Feb 5;24:63. doi: 10.1186/s12876-024-03148-4 (PMC10840143; doi:10.1186/s12876-024-03148-4)
Supplement: Supplementary file 1 — Additional file 1: Figure S1. Forest plot showing the results of multivariate logistic regression analysis 1. Figure S2. Forest plot showing the results of multivariate logistic regression analysis 2. Figure S3. Forest plot showing the results of multivariate logistic regression analysis 3. Figure S4. Forest plot showing the results of multivariate logistic regression analysis 4. Figure S5. Forest plot showing the results of multivariate logistic regression analysis 5. Figure S6. Forest plot showing the results of multivariate logistic regression analysis 6. Figure S7. Forest plot showing the results of multivariate logistic regression analysis 7. Figure S8. Forest plot showing the results of multivariate logistic regression analysis 8. Figure S9. Forest plot showing the results of multivariate logistic regression analysis 9. Figure S10. Forest plot showing the results of multivariate logistic regression analysis 10. [file 12876_2024_3148_MOESM1_ESM.docx]

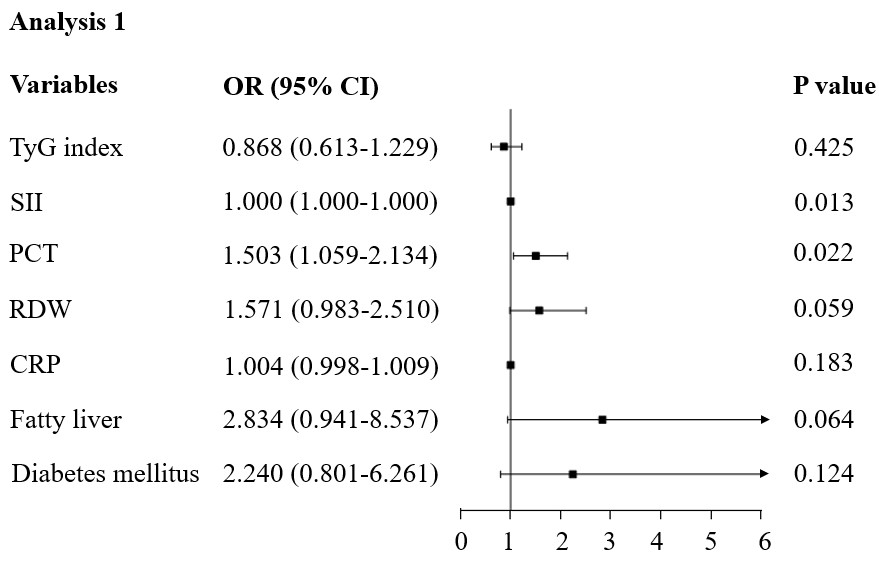


**Supplementary Figure 1:** Forest plot showing the results of multivariate logistic regression analysis 1.


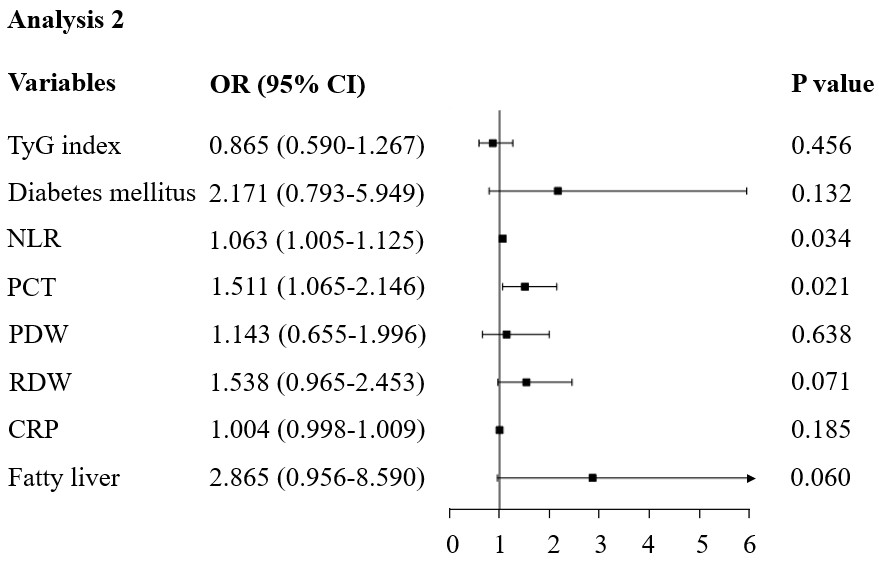


**Supplementary Figure 2:** Forest plot showing the results of multivariate logistic regression analysis 2.


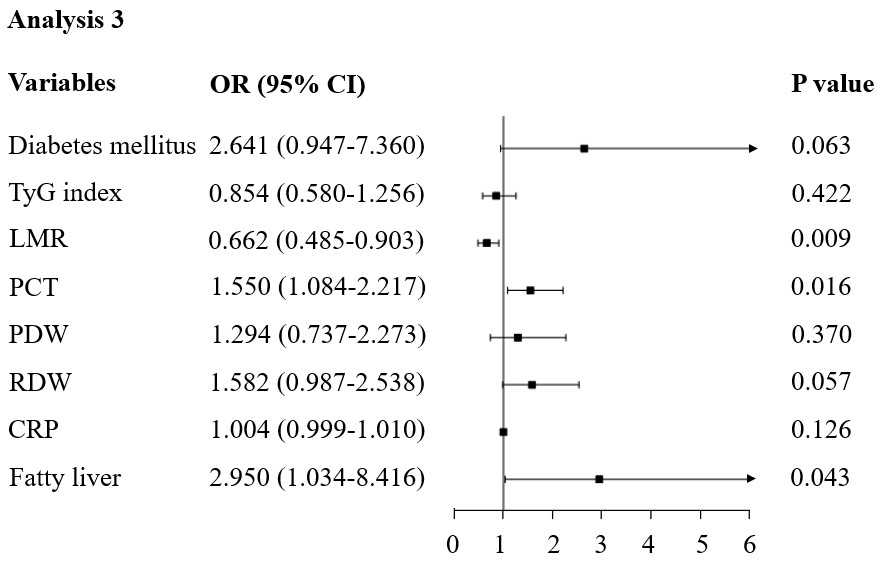


**Supplementary Figure 3:** Forest plot showing the results of multivariate logistic regression analysis 3.


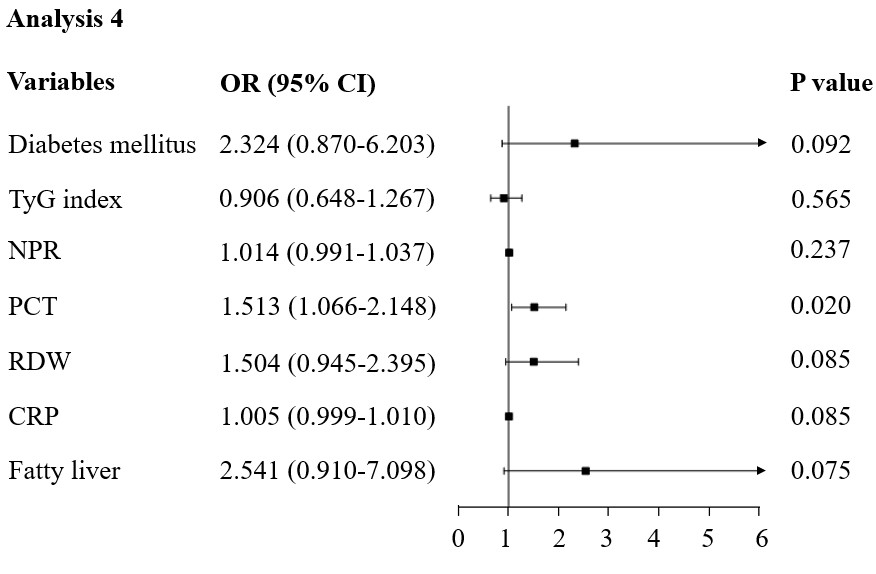


**Supplementary Figure 4:** Forest plot showing the results of multivariate logistic regression analysis 4.


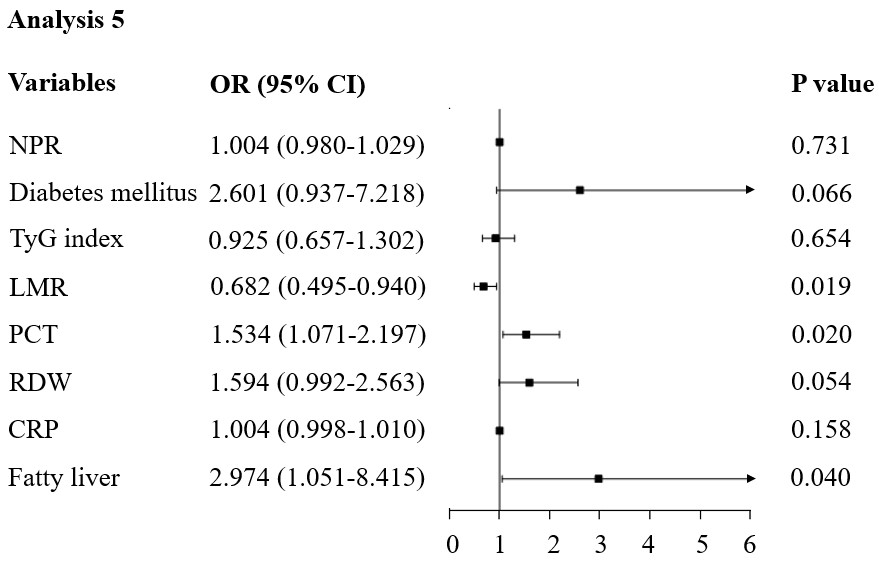


**Supplementary Figure 5:** Forest plot showing the results of multivariate logistic regression analysis 5.


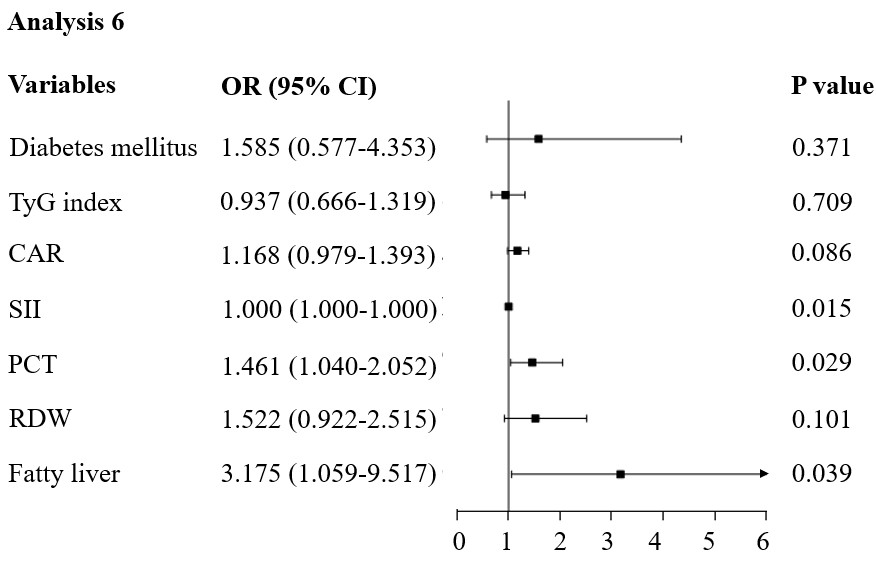


**Supplementary Figure 6:** Forest plot showing the results of multivariate logistic regression analysis 6.


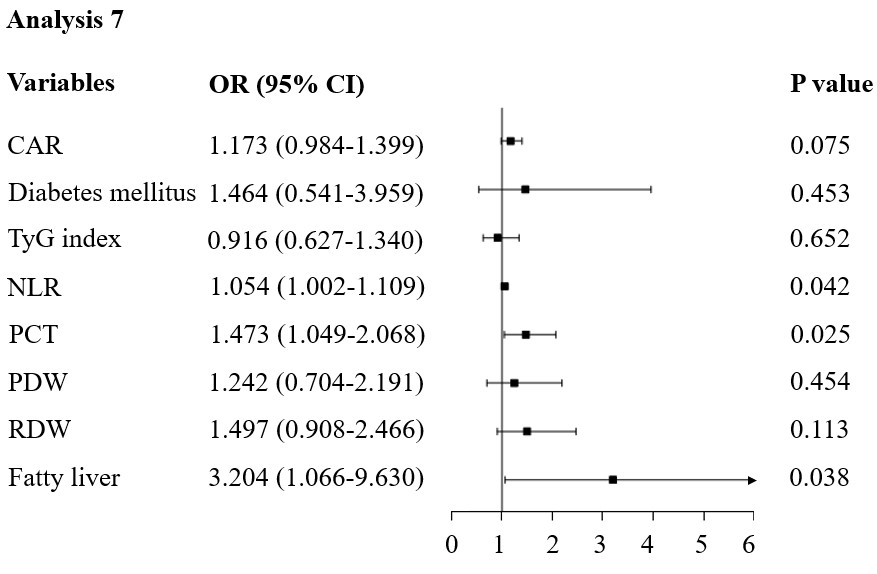


**Supplementary Figure 7:** Forest plot showing the results of multivariate logistic regression analysis 7.


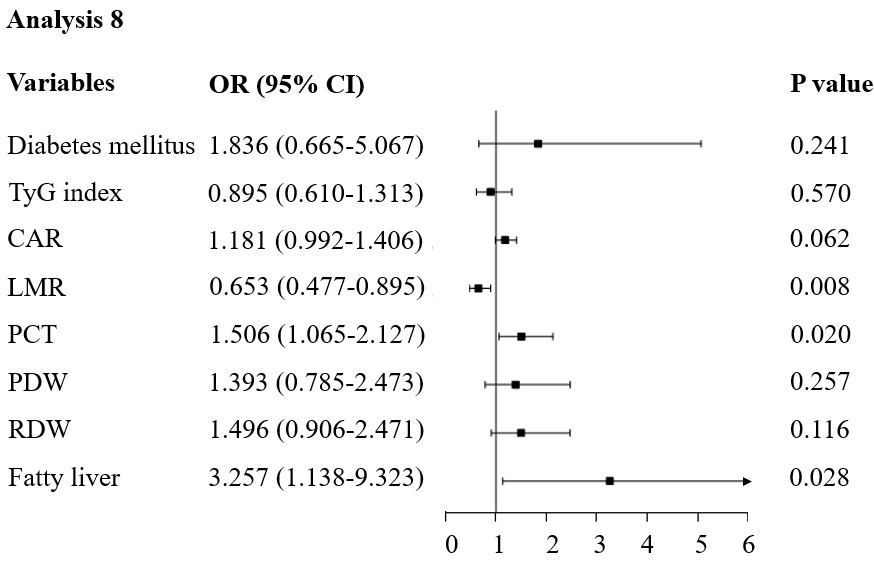


**Supplementary Figure 8:** Forest plot showing the results of multivariate logistic regression analysis 8.


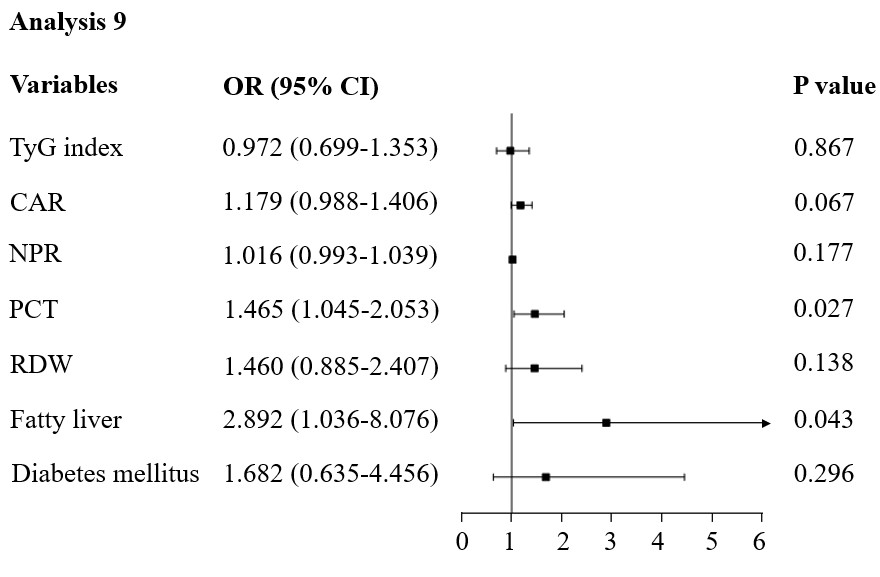


**Supplementary Figure 9:** Forest plot showing the results of multivariate logistic regression analysis 9.


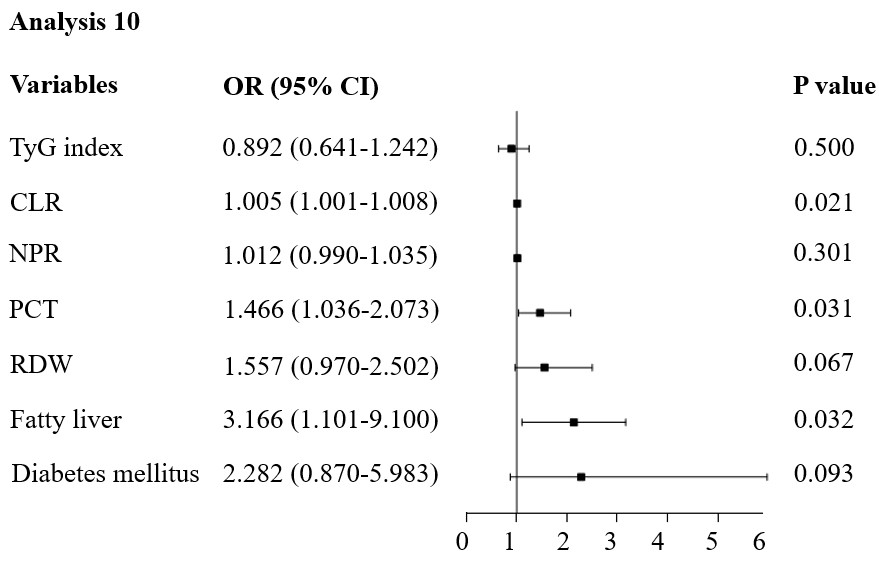


**Supplementary Figure 10:** Forest plot showing the results of multivariate logistic regression analysis 10.
